# Establishment of stable expression of firefly luciferase and EGFP in a canine inflammatory mammary carcinoma cell line and tumor-bearing model in nude mice

**DOI:** 10.3389/fvets.2022.935005

**Published:** 2022-08-02

**Authors:** Fangying Zhao, Xinqiu Li, Jiayue Liu, Di Zhang, Hongxiu Diao, Degui Lin

**Affiliations:** ^1^Department of Clinical Veterinary Medicine, College of Veterinary Medicine, China Agricultural University, Beijing, China; ^2^Key Laboratory of Animal Pathogen Infection and Immunology of Fujian Province, College of Animal Science (College of Bee Science), Fujian Agriculture and Forestry University, Fuzhou, China

**Keywords:** canine inflammatory mammary carcinoma, cell line, firefly luciferase, enhanced green fluorescent protein, characteristic, tumor-bearing model

## Abstract

Canine inflammatory mammary carcinoma (CIMC) is a type of canine malignant mammary tumor with a poor prognosis and high mortality. We transduced firefly luciferase and enhanced green fluorescent protein (EGFP) into CHMp, a CIMC cell line, and established CHMp-Luc-EGFP cells. We investigated the characteristics of this cell line *in vitro* and *in vivo*. CHMp-Luc-EGFP was passaged continuously 75 times, with stable expression of luciferase and EGFP. Compared with the wild-type, CHMp-Luc-EGFP had similar proliferation, metastasis, histopathology characteristics, and expression of E-cadherin, N-cadherin, and Ki-67. A tumor-bearing model was established by implantation of CHMp-Luc-EGFP cells, and the dynamic changes of tumors were visualized and quantified using the IVIS imaging system. In summary, the cell line we established could reflect the biological characteristics of CHMp cells, visualize the tumor progression *in vivo*, and provide a powerful tool for the study of CIMC.

## Introduction

Canine mammary tumors (CMTs) are the most prevalent types of tumors in female dogs. Most dogs with CMTs are not spayed, and the incidence rises with age (1), reaching ~60% in dogs between the ages of 8 and 13 years (2, 3). A study carried out in Italy showed an upward trend in female dogs having CMTs between 1985 and 2013 (2, 3): ~300 per 100,000 dog-years. The incidence is decreasing in some areas due to ovariohysterectomy carried out at a young age (4), but ~50% of CMTs are malignant (5), which is a huge threat to the life of dogs.

Canine inflammatory mammary carcinoma (CIMC) is a malignant type of CMT with poor differentiation, strong invasiveness, and high mortality. CIMC can be divided into primary and secondary types, but the common histopathological characteristic is an invasion of dermal lymphatic vessels by neoplastic emboli (6). The clinical features of CIMC are sudden presentation, edema, erythema, firmness, and warmth of the mammary glands, with or without mammary nodules (6–10). CIMC may be misdiagnosed clinically as dermatitis or mastitis due to the macroscopic inflammatory characteristics of the lesion (7, 10–12). Local or distant metastasis is found at the diagnosis (13). In addition to the usual sites of metastasis (e.g., lymph nodes and lungs), metastasis to the brain and spinal cord has also been documented (14). Surgical removal of CIMCs is difficult because of diffuse metastases, and CIMCs do not respond well to chemotherapy (15, 16) or radiotherapy (17). Uyama et al. (18) established CHMp, a CIMC cell line, to explore the metastatic mechanism of action of CIMCs (19, 20). To further investigate the invasiveness and metastasis of this cell line *in vivo*, we attempted a combination with *in vivo* imaging modalities.

*In vivo* imaging modalities have become valuable tools in immunology, oncology, virology, and neurology. Green fluorescent protein (GFP)-based fluorescence imaging was popular for several years. Subsequently, Zhang et al. (21) built an enhanced green fluorescent protein (EGFP), which was 35-times brighter and more sensitive than wild-type GFP. In recent decades, significant advances have been made in bioluminescence imaging (22). Bioluminescence imaging, in contrast to fluorescence measurements, provides higher sensitivity and lower background signals (23). In human medicine, cell lines transfected with GFP and luciferase have been used widely to establish tumor models at different depths (24).

Three proteins were selected to evaluate the capacity of cell lines for proliferation and metastasis. Ki-67 is a non-histone nuclear protein that has been explored as a biomarker for the proliferation and apoptosis of CMT cells (25–27). Epithelial (E)-cadherin and neural (N)-cadherin are calcium-dependent transmembrane glycoproteins that mediate cell-cell adhesion and modulate the migration and invasion of mammary tumor cells. The loss of E-cadherin and overexpression of N-cadherin are associated with increased invasive potential (28). As biomarkers of metastatic CMT, cadherins should be evaluated together with other biomarkers, such as Ki-67 (29, 30).

Despite the wide use of *in vivo* imaging methods and xenograft models, a commercially available CMT cell line or CIMC cell line for *in vivo* imaging is lacking. In addition, different cell lines have diverse characteristics, therapeutic effects, and molecular mechanisms of action. We aimed to establish a CIMC cell line that expressed luciferase and EGFP stably. We characterized it in terms of biological characteristics, tumorigenicity, histopathological characteristics, and protein expression. In this way, we provided an *in situ* tumor-bearing model and lung-metastasis model for subsequent studies of CIMC.

## Materials and methods

### Cell culture

A canine inflammatory mammary carcinoma cell line, CHMp, was kindly provided by the Graduate School of Agricultural and Life Sciences within the University of Tokyo (Tokyo, Japan). CHMp cells were cultured in Dulbecco's modified Eagle's medium (DMEM) supplemented with 10% fetal bovine serum (FBS), penicillin (100 U/ml), and streptomycin (100 μg/ml), all of which were from Life Technologies (Carlsbad, CA, USA), and incubated at 37°C in an atmosphere of 5% CO_2_.

### Establishment of a CHMp-Luc-EGFP cell line

Stable firefly luciferase-expressing and EGFP-expressing CHMp cells were developed by lentivirus (Ubi-MCS-EGFP-SV40-firefly_Luciferase-IRES-Puromycin; Shanghai Genechem. Shanghai, China), which expressed firefly luciferase, EGFP, and the puromycin-resistant gene. First, 6 × 10^4^ cells/well were cultured in 6-well cell-culture plates (Corning, Corning, NY, USA) and incubated for 24 h at 37°C, with replacement of the medium with complete DMEM containing the virus at the appropriate multiplicity of infection (MOI) and infection enhancer (MOI = 50). After 12 h of incubation, the medium was replaced with complete DMEM and cells were cultured continuously for 36 h. Transduced cells were selected in the presence of puromycin (3 μg/ml; Shanghai Genechem) for 3 days. Surviving cells were diluted to 10 cells/ml and seeded into 96-well cell culture plates (Corning) at 100 μl/well. Monoclonal cell lines were isolated after 10 days of culture. Transduction efficiency was detected by fluorescence microscopy using a CKX41 instrument (Olympus, Tokyo, Japan) and flow cytometry using a FACSCalibur^®^ system (BD Biosciences, San Jose, CA, USA).

### Fluorescence-activated cell detection

For flow cytometry, cells were collected, centrifuged at 155 × g for 5 min at room temperature, resuspended in phosphate-buffered saline (PBS; 500 μl; Solarbio Science & Technology, Beijing, China), and then passed through a cell strainer (70 μm; Biologix, Shandong, China). Next, 10,000 events were collected on the flow cytometer to assess transduction efficiency. FlowJo 10.7.1 (FlowJo, Ashland, OR, USA) was employed to analyze data.

### Luminescent intensity assay

An assay to measure luminescent intensity was undertaken to measure luciferase expression in CHMp-Luc-EGFP cells *in vitro*. Cells were seeded into 96-well plates (4, 3, 2, 1, 0.5, 0.25, or 0.125 × 10^4^ cells/well) and incubated for 24 h at 37°C. The bioluminescence intensity was measured using D-luciferin potassium salt (Fluorescence, Beijing, China) according to the manufacturer's instructions. Briefly, the medium was replaced with D-luciferin potassium salt (150 μg/ml), and luciferin activity was read immediately by the IVIS imaging system (PerkinElmer, Waltham, MA, USA). The fluorescence intensity was also detected by IVIS. Each group had three biological replicates.

### Assay to measure the proliferation and viability of cells

Cell Counting Kit 8 (CCK-8; Beyotime Institute of Biotechnology, Shanghai, China) was employed to assess the proliferation of cells *in vitro*. Briefly, 300 cells/well were seeded in 96-well plates and incubated at 37°C. CCK-8 (10 μl) was added to each well 1, 2, 3, 4, 5, or 6 days later. Following 50 min of incubation, the absorbance was read at 450 nm with a microplate reader (Agilent Technologies, Santa Clara, CA, USA). Each group had five biological replicates.

### Wound-healing assay

A wound-healing assay was carried out to analyze cell migration *in vitro*. Briefly, 2 × 10^5^ cells/well were seeded into six-well plates and incubated for 24 h at 37°C. A wound was created with a pipette tip. After washing two times with PBS to remove cell fragments, cells were maintained in DMEM with 2% FBS. Then, images of the wounds were photographed at 0 and 24 h to evaluate cell migration. Each group had three biological replicates.

### Invasion assay

An invasion assay was undertaken to assess the invasive ability of cells. Matrigel™ (BD Biosciences, San Jose, CA, USA) was mixed with DMEM at a ratio of 1:30. Then, we added Matrigel (100 μl) to the upper chamber of a 24-well Transwell™ plate (Labgic, Beijing, China), and incubation was allowed to proceed for 1 h at 37°C. Logarithmic-phase cells underwent trypsinization with 0.25% trypsin–EDTA (Life Technologies) and were collected with a serum-free medium. Next, 3 × 10^3^ cells/well were seeded into the upper chamber coated with the Matrigel membrane. Complete DMEM with 10% FBS was added to the lower chamber. After incubation for 24 h, non-invading cells were removed using a cotton swab. Invading cells were fixed with 4% paraformaldehyde and stained with 0.1% crystal violet (Solarbio Science & Technology). The fields of chambers were captured with a microscope (CKX41; Olympus) and invading cells were counted with ImageJ 1.53c (US National Institutes of Health, Bethesda, MD, USA). Each group had three biological replicates.

### Real-time reverse transcription-quantitative polymerase chain reaction

Gene expression of E-cadherin and N-cadherin was relatively quantified. Total RNA was extracted with the TransZol Up Plus RNA Kit (TransGen Biotech, Beijing, China) and reverse-transcribed with TransScript^®^ Uni All-in-One First-Strand cDNA Synthesis Supermix (TransGen Biotech) according to the manufacturer's instructions. Real-time PCR was carried out by the 7,500 Real-Time PCR system (Applied Biosystems, Carlsbad, CA, USA) and PerfectStart^®^ Green qPCR SuperMix (TransGen Biotech). Next, complementary DNA (0.1 μg) was added as a template to the qPCR reaction system (20 μl); the other components were the same as described in the manufacturer's instructions. Glyceraldehyde 3-phosphate dehydrogenase (GAPDH) was the reference gene. Each group had three biological replicates, and each biological replicate had three technical replicates.

Oligonucleotide primers (forward and reverse, respectively) were 5′-ATGTTTGTGATGGGCGTGAA-3′ and 5′-GCTAGAGGAGCCAAGCAGTT-3′ for GAPDH; 5′-TTCCTGCCATCTTGGGCATT-3′ and 5′-TGGCTCAAGTCAAAGTCCTGA-3′ for E-cadherin; and 5′-TAGCCCGGTTTCATTTGAGGG-3′ and 5′-ACTGTCCCGTTCCAAACCTG-3′ for N-cadherin.

### Western blotting

Cells were lysed using RIPA lysis buffer (Solarbio Science and Technology) with 1% phenylmethylsulfonyl fluoride (Solarbio Science and Technology) at 4°C. After 20 min, cells were collected with a scraper (Biologix). After centrifugation (13,201 × g, 15 min), lysates were collected and the protein concentration was measured with a bicinchoninic acid protein assay kit (Life Technologies). Protein samples were prepared following the addition of protein loading buffer (TransGen Biotech) and boiling. Total protein (12 μg) was separated by sodium dodecyl sulfate-polyacrylamide gel electrophoresis using 10% gels (Biotides, Beijing, China) and transferred to polyvinylidene difluoride (PVDF) membranes (Millipore, Merck, Darmstadt, Germany). PVDF membranes were blocked with 5% fat-free milk (Solarbio Science and Technology) for 1 h at room temperature and incubated with primary antibodies overnight at 4°C. Following incubation with horseradish peroxidase-conjugated secondary antibodies at 1:1,000 dilution (catalog number: ZB-2301 or ZB-2305; Zhong Shan-Golden Biological Technology, Beijing, China) for 1 h at 37°C, reactive bands were visualized under a chemiluminescent imaging system (5,200 series; Tanon Science and Technology, Beijing, China). The following primary antibodies were used at 1:1,000 dilution: E-cadherin (catalog number: 14472S, Cell Signaling Technology, Danvers, MA USA), N-cadherin (13116T; Cell Signaling Technology), and β-tubulin (M20005; Abmart, Shanghai, China). Each group had three biological replicates.

### Tumor models and *in vivo* imaging

Animal procedures were approved (AW62402202-2-1) by the Animal Ethics Committee of China Agricultural University (Beijing, China) according to guidelines for the care and use of laboratory animals set by the Chinese government.

To evaluate the tumorigenicity and stability of bioluminescence and EGFP of CHMp-Luc-EGFP *in vivo*, a tumor-bearing model was created. For the model of a primary tumor, 5 × 10^6^ cells were resuspended in PBS (100 l and injected (s.c.) into the left mammary fat pad of female BALB/c nude mice (5 weeks; 14 g; Vital River, Beijing, China). The weight and volume of tumors were monitored every day. For an experimental model of metastasis, 1 × 10^5^ cells were resuspended in PBS (100 μl) and injected into the tail vein. Weight was monitored every 3 days. At 1, 5, 10, 15, and 19 days after implantation, mice were injected with D-luciferin potassium salt (150 mg/kg, i.p.). Ten min later, mice were anesthetized with isoflurane (HFQ Bio-technology, Jiangsu, China), and luminescence and EGFP were visualized using the IVIS imaging system.

Mice were euthanized and tumor tissues harvested. The size and weight of tumors were measured to compare the tumorigenicity of the CHMp-Luc-EGFP cell line and CHMp cell line.

### Histopathology and immunohistochemistry

Tumor tissues and lung tissues were fixed with 10% neutral formalin (Solarbio Science and Technology) and embedded in paraffin (Solarbio Science and Technology). Then, tissues were cut serially into 5-μm-thick slices. Next, specimens were stained with H&E (Beyotime Institute of Biotechnology) at room temperature for histopathological characterization. For assessment of immunohistochemical characteristics, sections were incubated with anti-Ki-67 antibody (1:1,000; Proteintech, Rosemont, IL, USA).

### Statistical analyses

A two-tailed unpaired *t*-test was used to evaluate the differences between the two groups. Two-way ANOVA was used to compare the difference in tumor volume. *P* < 0.05 was considered significant. Results are the mean ± SD and were analyzed by Prism 8.0.1 (GraphPad, San Diego, CA, USA).

## Results

### Establishment of the CHMp-Luc-EGFP cell line

**Figure 1:**
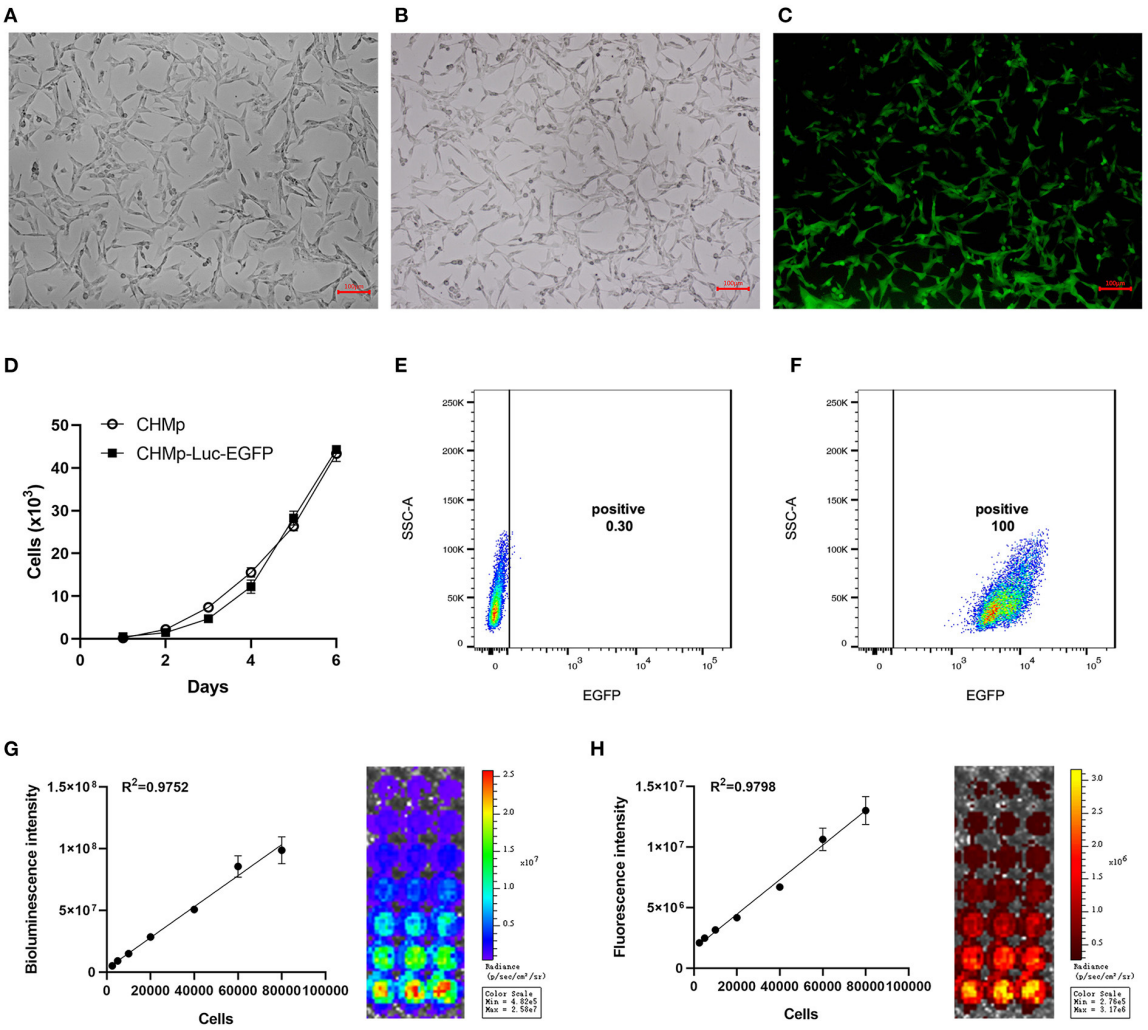
Establishment of the CHMp-Luc-EGFP cell line. **(A)** Morphological features of CHMp. **(B,C)** White-light and fluorescence images of CHMp-Luc-EGFP cells were captured. Magnification ×10; scale bar, 100 μm. **(D)** Growth curves of CHMp and CHMp-Luc-EGFP cell lines. Data are the mean of five repeat experiments. Each value is the mean ± SD. **(E,F)** EGFP expression of CHMp and CHMp-Luc-EGFP. **(G)** Bioluminescence of cells and correlation analysis between bioluminescence intensity and cell density. **(H)** Fluorescence of cells and correlation analysis between fluorescence intensity and cell density. Each value is the mean of three repeat experiments, mean ± SD.

Following transduction with lentivirus and selection with puromycin, nine monoclonal cell lines were obtained. The monoclonal cell line that showed the highest transduction efficiency (Figures 1B,C, Supplementary Figure 1), whose proliferation curve was most similar to that of the wild-type, was selected and named “CHMp-Luc-EGFP.” The morphological features of the cell lines were consistent (Figures 1A,B). The proliferation curve of CHMp-Luc-EGFP was similar to that of CHMp (Figure 1D).

Cells were cultured routinely. The transduction efficiency was monitored every 15 passages, which remained 100% at passage number 75 (Figures 1E,F). Therefore, CHMp-Luc-EGFP exhibited stable expression of luciferase and EGFP. The bioluminescence and fluorescence intensities of CHMp-Luc-EGFP were measured *in vitro*: the number of cells was strongly correlated with bioluminescence and fluorescence intensities (Figures 1G,H).

### Motility and invasion of CHMp-Luc-EGFP cells *in vitro*

**Figure 2:**
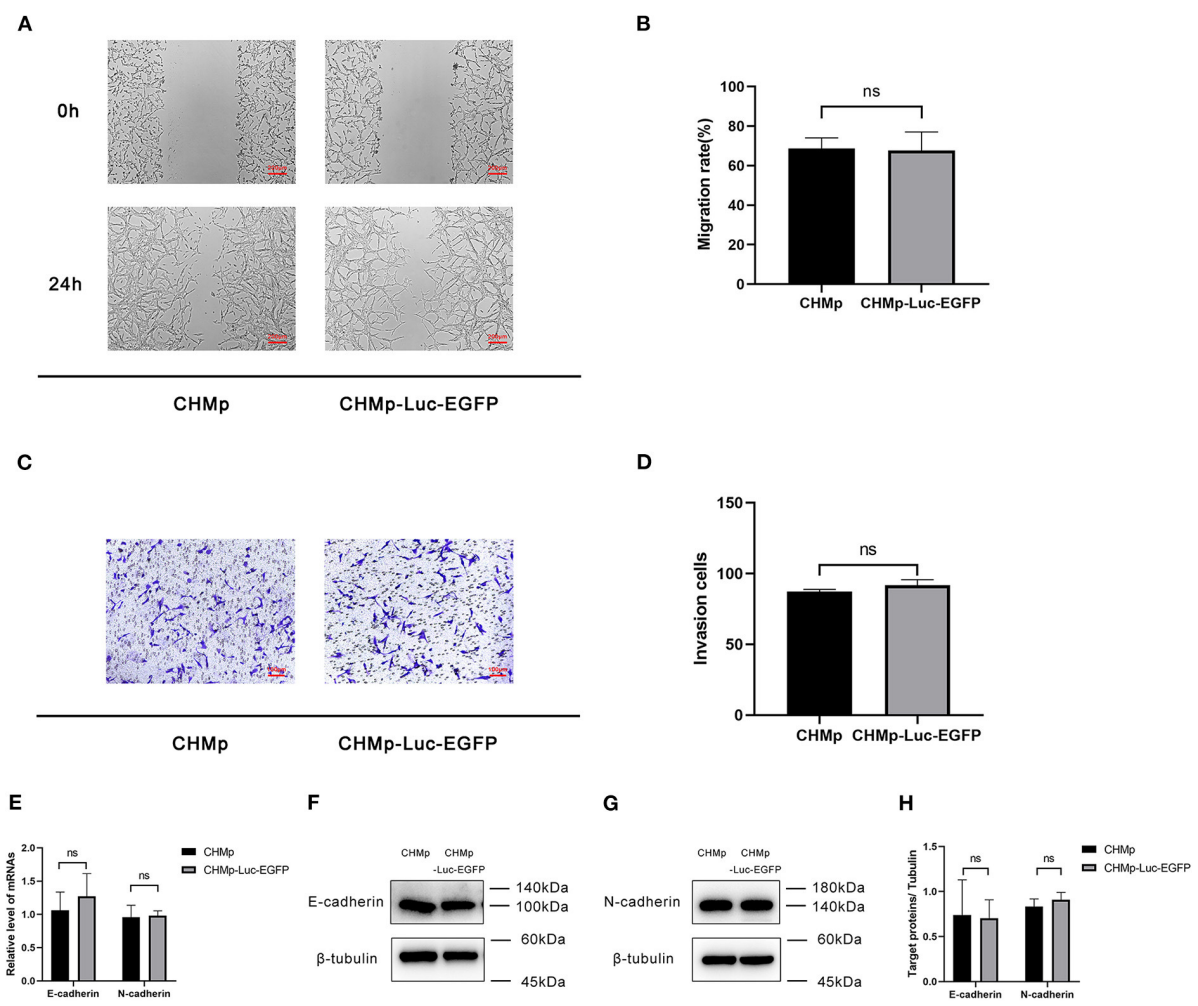
*In vitro* metastasis of CHMp-Luc-EGFP cells and CHMp cells. **(A,B)** Wound-healing assay. Percent migration of CHMp cells and CHMp-Luc-EGFP cells were calculated, which were 68.74% ± 5.33% and 67.68% ± 9.37%, respectively. Magnification ×4; scale bar, 200 μm. **(C,D)** Invasion assay. The number of invading cells was 87 ± 2 for CHMp compared with 92 ± 4 for CHMp-Luc-EGFP. Magnification ×10; scale bar, 100 μm. **(E)** mRNA expression of E-cadherin and N-cadherin. **(F–H)** Protein expression of E-cadherin and N-cadherin. Data are the mean of three independent experiments, mean ± SD. Significance was determined using a two-tailed unpaired *t*-test. ns, non-significant.

For the wound-healing assay, the migration of CHMP-Luc-EGFP cells and CHMp cells did not show a significant difference (Figures 2A,B). The number of invading cells was not significantly different according to the invasion assay (Figures 2C,D). E-cadherin and N-cadherin were relatively quantified to compare the migration potential and invasion potential of the cell lines: gene expression and protein expression of CHMp and CHMp-Luc-EGFP showed no significant difference (Figures 2E–H).

### Luminescent intensity of the CHMp-Luc-EGFP cell line *in vivo*

**Figure 3:**
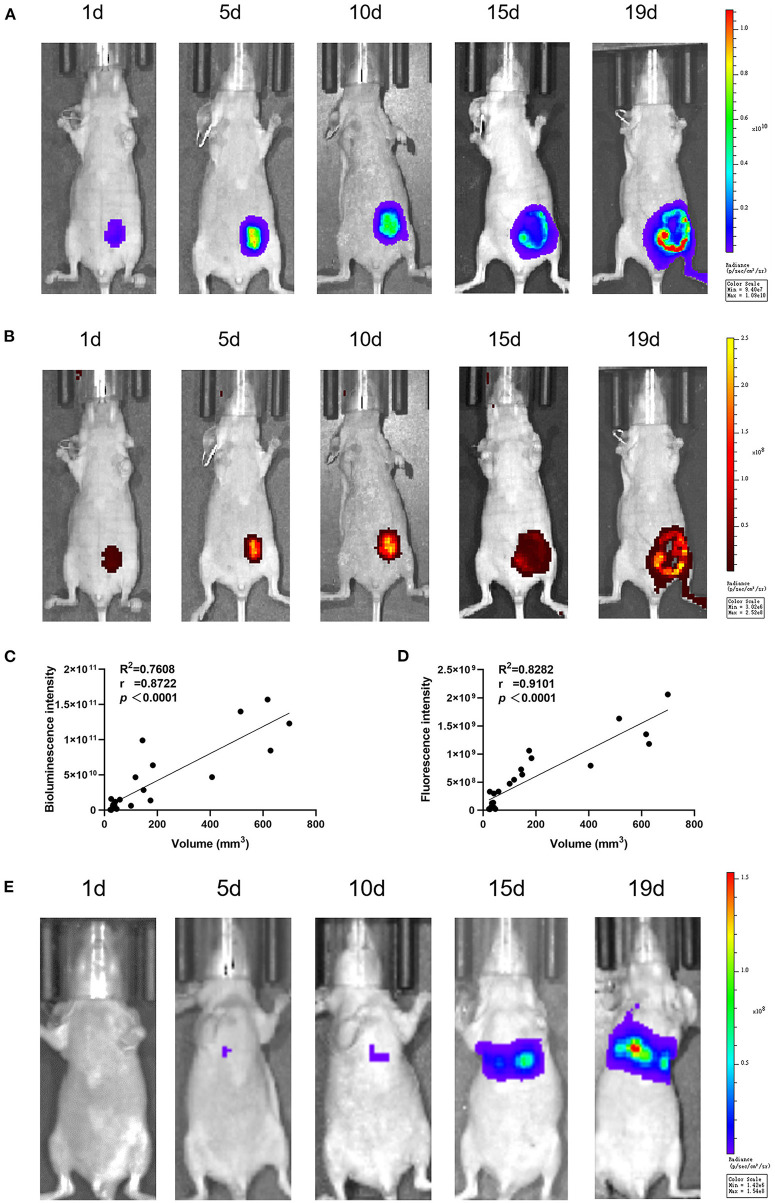
*In vivo* luminescent intensity of CHMp-Luc-EGFP cells. At 1, 5, 10, 15, and 19 days after implantation, luminescence and fluorescence images were captured with the IVIS imaging system. **(A)** Bioluminescence imaging of the primary-tumor model. **(B)** Fluorescence imaging of the primary-tumor model. **(C)** Correlation analysis between luminescent intensity and days after implantation. **(D)** Correlation analysis between luminescent intensity and tumor volume. **(E)** Bioluminescence imaging of the metastatic tumor model. *n* = 3.

The bioluminescence and fluorescence of CHMp-Luc-EGFP cells *in vivo* were photographed by the IVIS imaging system. For the primary-tumor model, the signal intensity became stronger on days 1, 5, 10, and 15, but there was a slight reduction on day 19 due to necrotic foci (Figures 3A,B). The luminescent intensity of the primary-tumor model became stronger after implantation, and the correlation between luminescent intensity and tumor volume was strong (Figures 3C,D). The bioluminescence intensity of the experimental model of metastasis became stronger after implantation (Figure 3E), but fluorescence signals could not be detected because the penetration depth of EGFP was not sufficient. In total, the proliferation and metastasis of cells *in vivo* could be reflected by the luminescent intensity.

### Proliferation and metastasis of CHMp-Luc-EGFP cells *in vivo*

**Figure 4:**
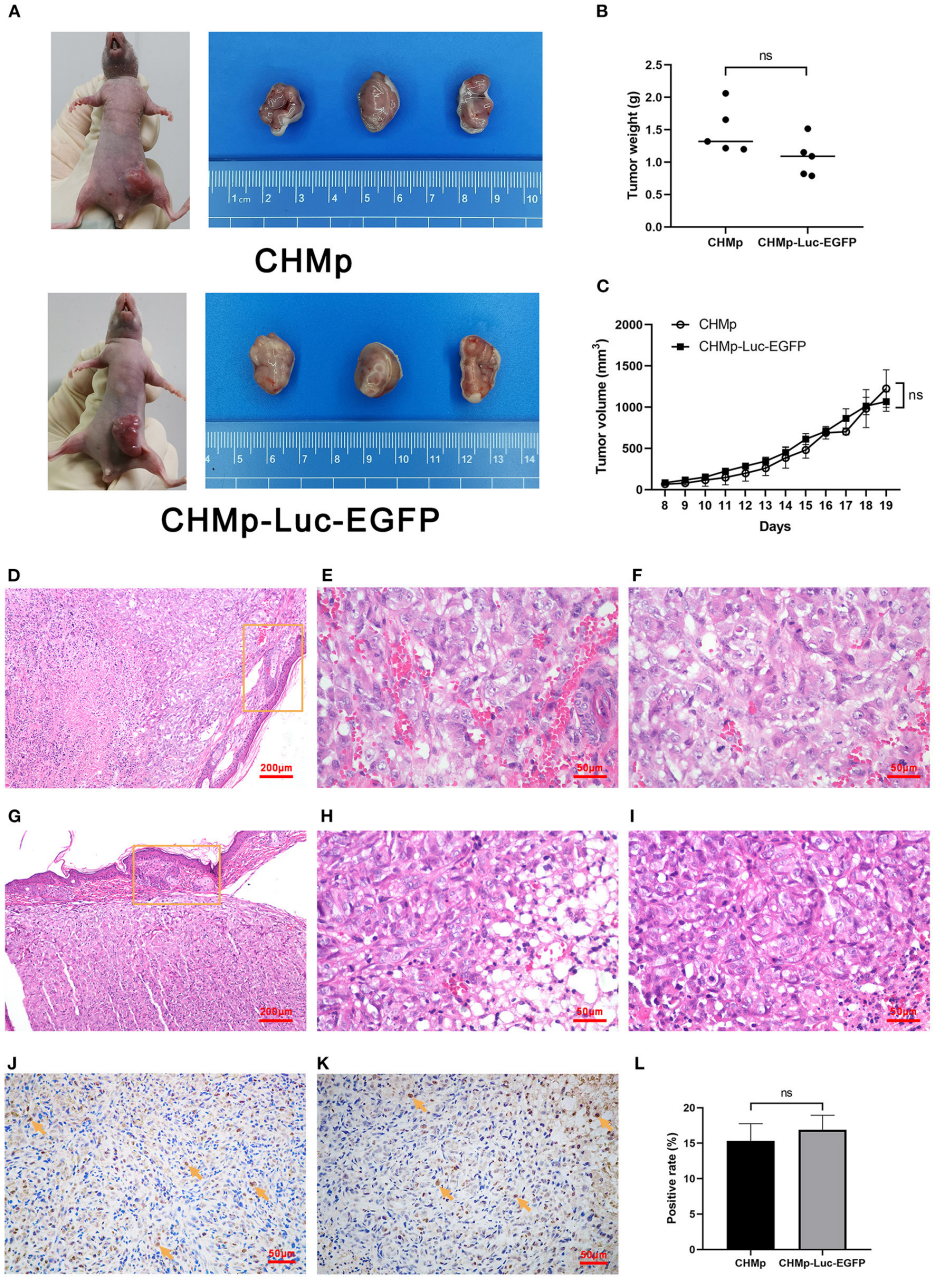
Tumorigenicity assay and histological sections of tumors derived from CHMp and CHMp-Luc-EGFP. **(A)** The volume of subcutaneously transplanted tumors. **(B)** Weight of subcutaneously transplanted tumors. Significance was determined using a two-tailed unpaired *t*-test; ns, non-significant. *n* = 5. **(C)** Changing sizes of subcutaneously transplanted tumors in each group over time. Significance was determined using two-way ANOVA. *n* = 5. **(D–I)** Pathological and immunohistochemical sections of tumors were derived from CHMp (upper panels) and CHMp-Luc-EGFP (lower panels). The circled area showed skin invasion. **(D,G)** Magnification ×10; scale bar, 200 μm. **(E–I)** Magnification ×40; scale bar, 50 μm. **(J,K)** Immunohistochemical sections of tumors were derived from CHMp cells (left) and CHMp-Luc-EGFP cells (right). Positive cells were indicated with arrows. Magnification ×40; scale bar, 50 μm. **(L)** Percentage of Ki-67-positive CHMp cells and Ki-67-positive CHMp-Luc-EGFP cells was 15.31% ± 2.45% and 16.89% ± 2.05%, respectively. Data are the mean of three regions and are presented as the mean ± SD. Significance was determined using a two-tailed unpaired *t*-test; ns, non-significant.

A tumor-hearing model was generated to assess cell proliferation *in vivo*. The volume and weight of tumor tissues in mice were not significantly different when the two cell lines were used (Figures 4A–C). The pathologic histology grading of tumor masses derived from CHMp cells and CHMp-Luc-EGFP cells was grade 2. In specimens, there were many poorly differentiated cells, with obvious cell atypia (Figures 4D–I). Skin invasion was seen in specimens. Immunohistochemical sections showed that the number of Ki-67-positive cells was not different between the two cell lines (Figures 4J–L, Supplementary Figures 2A,B).

## Discussion

We established a CIMC cell line with firefly luciferase and EGFP. CIMC is a rare form of CMT that accounts for only 7.6% of dogs examined for dysplasia or tumors of the mammary glands (11). However, CIMC is characterized by rapid progression, aggressive behavior, high metastasis, and high mortality. More than 80% of dogs with CIMC have local or distant metastasis at the diagnosis (11, 13). Case reports have suggested that CIMC can also metastasize to the spinal cord and brain (14), making CIMC harder to treat. The median duration of survival is ~6 months (31), and appropriate treatment plans are lacking (17, 32, 33). Trimodal treatment (chemotherapy, surgery, and radiotherapy) is recommended for inflammatory breast cancer in humans (34), which could be the direction of CIMC treatment. Thus, we established cell line and xenograft models for a deeper evaluation of the efficacy of different therapies.

Due to the aggressive behavior and high metastasis rate of CIMC, creating a cell line that can be used for *in vivo* imaging is a rational approach, but which imaging method should be selected? The readouts of fluorescence and bioluminescence are different in several aspects. The advantage of fluorescent proteins such as EGFP is that they are substantially brighter than bioluminescent proteins. Increasing the amount of exciting light can make it brighter, whereas the intensity of bioluminescence is strictly limited by the number of substrates catalyzed by luciferase. Because of its low brightness, bioluminescence requires a longer exposure time. Conversely, bioluminescence signals do not require external excitation light, so they usually have a higher signal-to-noise ratio and are more sensitive than fluorescence signals, which results in a lower background signal (23, 35–37).

According to the discussion above, CHMp-Luc-EGFP was transduced with EGFP and firefly luciferase to suit different needs. We could see the transduction efficiency clearly and intuitively with fluorescence, but a stable expression of EGFP did not mean that luciferase could also be expressed stably, so we carried out luciferase detection in addition. Bioluminescence signals may be more sensitive in the experimental model of metastasis. Due to the lack of penetration depth, fluorescence imaging could detect only relatively superficial signals. The correlation analysis between tumor size *in vivo* and signal intensity showed a higher degree of linear fitting, which indicated that the conventional *in vivo* tumor-volume formula may have some deviation for estimation of tumor depth. The formula needs to be optimized further, and *in vivo* imaging can provide an auxiliary reference for the assessment of tumor volume.

Although histopathological findings did not show obvious lymphatic invasion, skin invasion was seen, which was confirmed by bioluminescence imaging. Therefore, we considered this cell line to be CIMC. The underlying reason for the absence of lymphatic invasion was that the tumor grew so rapidly that its size had exceeded 1,500 mm^3^ before significant metastasis occurred. The deficiency of the immune system and absence of lymphatic vessels in nude mice, and the impact of species differences, were potential factors.

Ki-67, E-cadherin, and N-cadherin were selected as biomarkers to compare CHMp and CHMp-Luc-EGFP. Ki-67 is detected mainly in proliferating cells. It is visible in the G1 phase of the cell cycle, and gets stronger as the cell cycle progresses to the S phase and G2 phase. Ki-67 expression is maximal during the M phase, and decreases rapidly after mitosis (29). Immunohistochemical staining of Ki-67 is more precise than measuring the mitotic index with histological staining using H&E because the mitotic index displays only the number of cells during mitosis, whereas Ki-67 is produced in different phases of the cell cycle (27).

E-cadherin is a major component of adherent junctions in normal epithelial cells and many types of carcinoma cells, where it acts as a suppressor of the motility and invasion of cells (38). N-cadherin is a mesenchymal marker in epithelial–mesenchymal transformation (EMT). Abnormal expression of N-cadherin is closely related to the invasion and metastasis of tumor cells (39). The switch from E-cadherin to N-cadherin is often observed in aggressive cancers (40). Expression of Ki-67, E-cadherin, and N-cadherin indicated that CHMp cells and CHMp-Luc-EGFP cells were in EMT with a high degree of malignancy.

We demonstrated that CHMp-Luc-EGFP cells and CHMp cells had similar biological characteristics in terms of proliferation, migration, tumorigenicity, and protein expression. Based on bioluminescence imaging and fluorescence imaging, the newly established cell line and xenograft models could be used widely in localization or quantitative studies, such as monitoring the growth and metastasis of CIMC cells and treatment efficacy, which can provide more reliable data for research in small animals.

## Conclusions

We established a CIMC cell line that expressed luciferase and EGFP stably. We compared differences in cell characteristics, tumorigenicity, histopathology, and protein expression between CHMp-Luc-EGFP and the wild-type, thereby providing a basis for subsequent transformation of other CMT cell lines. Based on the strong aggressiveness and high metastasis rate of CIMC, we established an *in situ* tumor-bearing model and lung-metastasis model in nude mice to visualize and quantify the effect of treatments against tumors.

## Data availability statement

The original contributions presented in the study are included in the article/Supplementary material, further inquiries can be directed to the corresponding author/s.

## Ethics statement

The animal study was reviewed and approved by Animal Ethics Committee of China Agricultural University.

## Author contributions

FZ and XL carried out the experimental work, data analyses, and wrote the manuscript. JL took part in some experiments. HD designed the entire study. HD and DZ revised the manuscript. DL contributed to conceptualization and funding acquisition. All authors approved the submitted version of this manuscript.

## Funding

This study was supported by the Research Fund (Clinical Diagnosis and Treatment of Pet) for Young College Teachers in Ruipeng Commonweal Foundation (RPJJ2020019), Innovation Training Program for College Students of Fujian Province (202110389056 and X202210389076), and the Educational Research Project for Young and Middle-aged Teachers of Fujian Provincial Department of Education (JAT210085).

## Conflict of interest

The authors declare that the research was conducted in the absence of any commercial or financial relationships that could be construed as a potential conflict of interest.

## Publisher's note

All claims expressed in this article are solely those of the authors and do not necessarily represent those of their affiliated organizations, or those of the publisher, the editors and the reviewers. Any product that may be evaluated in this article, or claim that may be made by its manufacturer, is not guaranteed or endorsed by the publisher.

## Supplementary material

Supplementary Figure 1 Fluorescence image of CHMp cells. Magnification ×10; scale bar, 100 μm.

Supplementary Figure 2 Ki-67 negative control of immunohistochemical sections of tumors derived from CHMp cells **(A)** and CHMp-Luc-EGFP cells **(B)**. Magnification ×40; scale bar, 50 μm.
