# Supplementary figures and images for: Establishment of stable expression of firefly luciferase and EGFP in a canine inflammatory mammary carcinoma cell line and tumor-bearing model in nude mice
Source: Front Vet Sci. 2022 Aug 2;9:935005. doi: 10.3389/fvets.2022.935005 (PMC9378969; doi:10.3389/fvets.2022.935005)

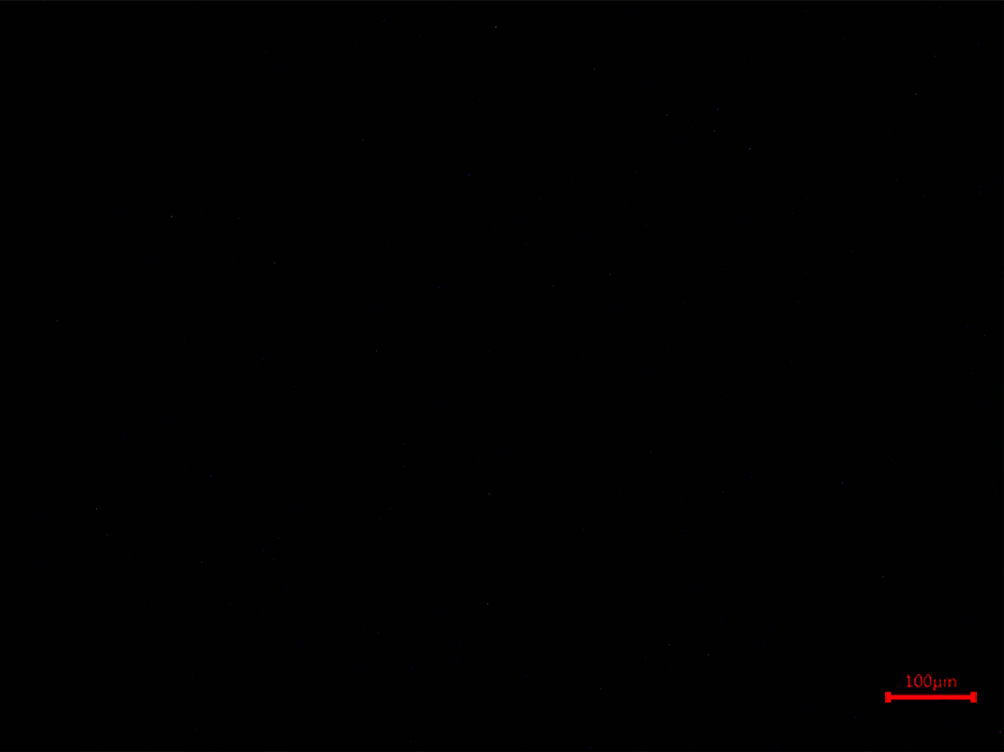

Supplement: Supplementary Figure 1 — Fluorescence image of CHMp cells. Magnification ×10; scale bar, 100 μm. [file Image_1.JPEG]

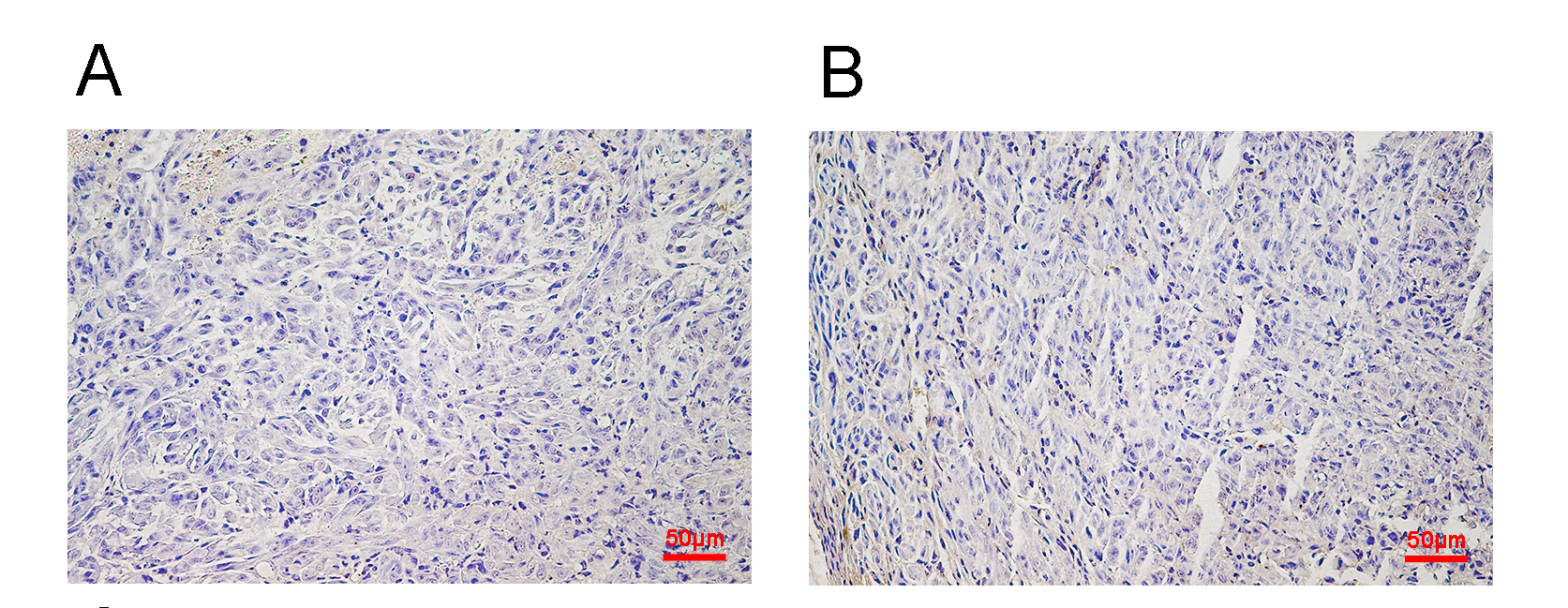

Supplement: Supplementary Figure 2 — Ki-67 negative control of immunohistochemical sections of tumors derived from CHMp cells (A) and CHMp-Luc-EGFP cells (B). Magnification ×40; scale bar, 50 μm. [file Image_2.JPEG]
